# The Ear

**Published:** 1871-09

**Authors:** 


### THE EAR.

The first institution established in this country for the exclusive treatment of diseases of the ear, especially for the benefit of the poor and those who can pay but little, is in West Thirty-fifth Street, and is called The New York Ear Dispensary, where everything will be done which medical skill can devise for the relief of the suffering; and it is earnestly hoped that a benevolent public will sustain this institution by liberal contributions.

